# Immune Regulatory Genes Are Major Genetic Factors to Behcet Disease: Systematic Review

**DOI:** 10.2174/1874312901812010123

**Published:** 2018-08-29

**Authors:** Yan Deng, Weifeng Zhu, Xiaodong Zhou

**Affiliations:** 1 The Second Affiliated Hospital of Nanchang University, Nanchang, China; 2 Department of Internal Medicine/Rheumatology, University of Texas Health Science Center at Houston McGovern Medical School, Houston, USA; 3 College of Basic Medical Sciences, Nanchang University, Nanchang, China

## Immune Regulatory Genes Are Major Genetic Factors to Behcet Disease: Systematic Review

The Open Rheumatology Journal, **2018**, 12: 70-85

The revised article type is mentioned below:

**Systematic Review**

The original article type provided was: 

**Review Article**

The revised affiliation is mentioned below:

**Department of Internal Medicine/Rheumatology, University of Texas Health Science Center at Houston McGovern Medical School, Houston, USA**

The original affiliation provided was:

**Department of Internal Medicine/Rheumatology, University of Texas Health Science Center at Houston McGovern Medical School, USA**

